# Genomic Prediction of Biological Shape: Elliptic Fourier Analysis and Kernel Partial Least Squares (PLS) Regression Applied to Grain Shape Prediction in Rice (*Oryza sativa* L.)

**DOI:** 10.1371/journal.pone.0120610

**Published:** 2015-03-31

**Authors:** Hiroyoshi Iwata, Kaworu Ebana, Yusaku Uga, Takeshi Hayashi

**Affiliations:** 1 Department of Agricultural and Environmental Biology, Graduate School of Agricultural and Life Sciences, University of Tokyo, Bunkyo, Tokyo, Japan; 2 Genetic Resources Center, National Institute of Agrobiological Sciences, Tsukuba, Ibaraki, Japan; 3 Agronomics Research Center, National Institute of Agrobiological Sciences, Tsukuba, Ibaraki, Japan; 4 Agroinformatics Division, National Agricultural Research Center, National Agriculture and Food Research Organization, Tsukuba, Ibaraki, Japan; Meiji University, JAPAN

## Abstract

Shape is an important morphological characteristic both in animals and plants. In the present study, we examined a method for predicting biological contour shapes based on genome-wide marker polymorphisms. The method is expected to contribute to the acceleration of genetic improvement of biological shape via genomic selection. Grain shape variation observed in rice (*Oryza sativa* L.) germplasms was delineated using elliptic Fourier descriptors (EFDs), and was predicted based on genome-wide single nucleotide polymorphism (SNP) genotypes. We applied four methods including kernel PLS (KPLS) regression for building a prediction model of grain shape, and compared the accuracy of the methods via cross-validation. We analyzed multiple datasets that differed in marker density and sample size. Datasets with larger sample size and higher marker density showed higher accuracy. Among the four methods, KPLS showed the highest accuracy. Although KPLS and ridge regression (RR) had equivalent accuracy in a single dataset, the result suggested the potential of KPLS for the prediction of high-dimensional EFDs. Ordinary PLS, however, was less accurate than RR in all datasets, suggesting that the use of a non-linear kernel was necessary for accurate prediction using the PLS method. Rice grain shape can be predicted accurately based on genome-wide SNP genotypes. The proposed method is expected to be useful for genomic selection in biological shape.

## Introduction

Shape is an important morphological characteristic in animals and plants [1]. Shapes of plant organs such as leaves, flowers, and seeds, are key taxonomic characteristics used to classify plant species. In dietary plants, the organ shape is an important characteristic related to the quality and quantity of agricultural products. Therefore, it has remained an important target in plant breeding [2]. For example, in cereal crop plants, grain shape is an important trait that is related to the intended end-usage, consumer’s preference [3], and processing properties such as milling quality [4, 5], [6]. Actually, rice grain shape shows wide variation across countries, stemming in part from differences among traditional varieties in numerous local regions [7, 8]. Shape varies to such a degree that it affects consumer preferences, influencing the market shares of the respective varieties [9, 10]. Because of its importance, the genetic variation in grain shape has been elucidated using bi-parental quantitative trait locus (QTL) mapping [10–12] and association mapping [13]. Detected QTLs have not been used efficiently in practical breeding, with few exceptions [14], mainly because the grain shape is a quantitative trait controlled by several genes that often have complex pleiotropic effects [10]. Tracking a small number of these through DNA markers will only explain a small fraction of the genetic variance [15].

A new breeding method for improving quantitative traits, designated as genomic selection (GS) [16], has increasingly attracted attention in animal [15, 17] and plant [18–20] breeding. Most importantly, GS is a technology for selecting individuals and lines without measuring phenotypic data. Instead the genomic potential of individuals and lines is predicted based on genome-wide marker polymorphisms. Then selection is performed on the predicted genetic potential (i.e., genomic prediction). Genome-wide marker polymorphisms can be examined in the early growth stage. Therefore, combined use of GS and rapid generation advancement is expected to boost genetic improvement of domesticated animals [15] and crop plants [18, 21]. Because GS uses numerous markers distributed over the whole genome, it is known to be suitable for the improvement of quantitative traits controlled by numerous QTL. Consequently, GS is expected to be an efficient method for the genetic improvement of quantitative traits.

Motivated by a sense of expectation to the potential of GS in plant and animal breeding, genomic prediction has been applied to various quantitative traits by way of trials and evaluations of its accuracy [22–24]. Genomic prediction, however, has been applied mainly to low-dimensional traits. Few attempts have been made to apply genomic prediction to a trait measured as high-dimensional data, such as biological contour shape. Even for simple contour shapes, as those of rice grains, its variation is represented by displacement in the *x* and *y* coordinates of grain contours, and show continuous differences among varieties and lines because of the influence of multiple genes that are responsible for grain shape variation and environmental factors that affect the grain shape. Elliptic Fourier (EF) analysis [25], an efficient method for measuring contour shape variations, has been applied to various biological shape analyses in plants and animals [1, 26]. In the EF analysis, a biological contour shape is described mathematically by Fourier series expansions. Then the coefficients of the Fourier expansions are used as shape descriptors: elliptic Fourier descriptors (EFDs). An important benefit of this method is the ease of interpretation of the analysis results via the visualization of shape variations. For example, the morphological influence of QTL can be represented visually [13]. The visualization ability can be an extremely useful feature in the application of the EF analysis to the genomic prediction of a biological contour shape.

In this study, we predict the biological contour shape based on genome-wide single nucleotide polymorphisms (SNPs). As a proof-of-concept study, we applied the method to the prediction of rice (*Oryza sativa* L.) grain shape variations. A contour shape of brown rice grain was delineated by EFDs. Then the EFDs were predicted based on genome-wide marker polymorphisms. We built prediction models using four methods: linear or nonlinear and single-dimensional or multi-dimensional regression methods. We compared the accuracy of the methods using multiple datasets of rice germplasm collections, which had different characteristics of marker density and sample size. The objectives of this study were (1) to propose a method for predicting rice grain shape delineated by EFDs based on genome-wide marker polymorphisms, (2) to assess the accuracy of the genomic prediction of rice grain shape, and (3) to ascertain an appropriate method for building a model for predicting rice grain shape. We then discussed the potential of the proposed method for genomic selection of the biological contour shape.

## Materials and Methods

### Rice germplasm collections and their marker genotypes and grain images

We used two independent datasets to assess the potential of genomic prediction of rice grain shape. The first one, dataset A, included 179 of the 332 rice accessions that had been selected as representatives of the rice germplasm at the National Institute of Agrobiological Sciences (NIAS) Genebank [27]. The second one, dataset B, contained 386 of the 395 rice accessions that had been used in a study by Zhao et al. [28] to assess genome-wide patterns of polymorphism, population structure, and the introgression history of *O*. *sativa*. We also analyzed the third one, dataset C, which was a subset of dataset B, used by Zhao et al. [29] to conduct a genome-wide association study using diverse accession of *O*. *sativa*. We used genome-wide SNP marker data and grain image data of the 386 accessions that were available to the public at http://ricediversity.org/. Information related to the accessions included in datasets A, B and C is presented in S1–S2 Tables.

Genome-wide SNP marker data for datasets A, B, and C were collected as described below. For dataset A, the 179 accessions were genotyped with 3,254 SNPs as described by Yamamoto et al. [30], although subsets of the accessions were not typed with all markers: 90 accessions were typed with a set of 3,194 SNPs, 17 with a set of 768 SNPs, and 96 with a set of 765 SNPs. Missing and untyped SNP genotypes were imputed using fastPHASE ver. 1.3 [31] by following the procedures described by Iwata and Jannink [32]. The imputation step was repeated 100 times. The mean imputation scores were obtained by averaging imputed genotypes over the 100 replications, as proposed by Iwata and Jannink [32]. For datasets B and C, we used genome-wide SNP data that were available at http://ricediversity.org/data/, as described above. For dataset B, we analyzed the genotype data of the 1,311 SNPs that had been genotyped and analyzed by Zhao et al. [28]. For dataset C, we analyzed the genotype data of the 36,901 SNPs that had been genotyped and analyzed by Zhao et al. [29]. For both datasets, we imputed missing genotypes using fastPHASE in the same way as dataset A. The proportions of imputed genotypic data were 47.3%, 3.1%, and 4.3%, respectively, for datasets A, B, and C.

Brown rice grain images of the 179 and 386 accessions were collected, respectively, for datasets A and B. For dataset A, we used the EFD data of the 179 accessions, which had been analyzed an earlier study [13]. The EFDs of six grains were measured and recorded for each accession. For dataset B, we analyzed the digital images of brown rice grains, which were downloaded from http://ricediversity.org/photolibrary/. The images of four grains were available for each accession. We measured the EFDs of the rice grains using the procedure described in the following section. As described above, dataset C was a subset of dataset B. Therefore, the phenotypic data collected for dataset B were used also for dataset C.

The datasets used in this study are annexed in S1 Dataset.

### Quantitative description of rice grain shape via EFDs

A quantitative description of the rice grain shape was conducted as described in Iwata et al. [13] using the SHAPE program package [33]. Rice grain contours were extracted using the digital image analysis of the rice grain images. An extracted contour of each grain was represented as a sequence of *x* and *y* coordinates of boundary pixels on the contour. Assuming the *x* and *y* coordinates of the pixel at the length of the contour *t* from the arbitrary starting pixel, i.e., *x*(*t*) and *y*(*t*), as the coordinates of a particle travelling around the contour at a constant speed 1, the variations of *x* and *y* coordinates became periodic functions of *t* and were approximated with Fourier series as

$$x(t)=a_{0}+\sum_{n=1}^{N}\left(a_{n}\text{cos}\frac{2n\pi t}{T}+b_{n}\text{sin}\frac{2n\pi t}{T}\right)$$ (1)

and

$$y(t)=c_{0}+\sum_{n=1}^{N}\left(c_{n}\text{cos}\frac{2n\pi t}{T}+d_{n}\text{sin}\frac{2n\pi t}{T}\right)$$ (2)

where *a*_*n*_, *b*_*n*_, *c*_*n*_, and *d*_*n*_, are Fourier coefficients of the *n*th harmonic. *a*_0_ and *c*_0_ merely depend on the position of the contour and is unrelated to the shape of the contour. Therefore, we ignored both coefficients in the following analysis (i.e., replacing both coefficients with 0). In this study, we approximated the contour coordinates of boundary pixels on the contour of a rice grain by the Fourier series with the first 20 harmonics (i.e., *N* = 20). Error in the Fourier series approximation with the first *N* harmonics was calculated as the proportion of squared displacements between observed and approximated contour coordinates to the total sum of squares of the variations of observed contour coordinates.

$$E_{N}^{2}=\sum_{p=1}^{P}\frac{{\left(x_{p}-x_{Np}\right)}^{2}+{\left(y_{p}-y_{Np}\right)}^{2}}{{\left(x_{p}-\bar{x}\right)}^{2}+{\left(y_{p}-\bar{y}\right)}^{2}}$$

Therein, *x*_*p*_ and *y*_*p*_ are the observed contour coordinates (*p* = 1, 2, …, *P*), $\bar{x}=\sum_{p=1}^{P}x_{p}$ and $\bar{y}=\sum_{p=1}^{P}y_{p}$ are the coordinates of the centroid of each contour, and *x*_*Np*_ and *y*_*Np*_ are the approximated coordinates, respectively corresponding to *x*_*p*_ and *y*_*p*_. The numbers of pixels (*P*) differed between grains, depending on the size of grains and the scale of grain images. For this study, we evaluated displacements between the observed and approximated coordinates equally over the entire contour. If one were interested in local shape variation, then one could assign a larger weight to squared displacements for the region of interest than to the remainder.

Because the Fourier coefficients, *a*_*n*_, *b*_*n*_, *c*_*n*_, and *d*_*n*_, calculated as described above were not invariant to size, rotation, and the position of a starting point of the contour trace, they were standardized to be invariant to these factors according to the size and direction of the long axis of the first harmonic ellipse [25, 34]. The standardization can be performed mathematically (i.e., in an objective manner). It has been used in quantitative genetics analysis of the biological shape [13, 35–38]. After this standardization, three Fourier coefficients became constant (*a*_1_ = 1, *b*_1_ = 0, *c*_1_ = 0). The remaining 4*N* – 3 coefficients were used as “descriptors” of shape. Hereinafter, we use vector notation $f={\left(a_{-1}^{\text{T}},b_{-1}^{\text{T}},c_{-1}^{\text{T}},d^{\text{T}}\right)}^{\text{T}}={\left(a_{2},\ldots ,a_{N},b_{2},\ldots ,b_{N},c_{2},\ldots ,c_{N},d_{1},\ldots ,d_{N}\right)}^{\text{T}}$ to represent the standardized EFDs, where subscript “–1” denotes the exclusion of the first harmonic coefficients *a*_1_, *b*_1_, and *c*_1_ because they became constant through the standardization procedure [25]. We set *N* as 20 in the present study. Therefore the dimensionality of the vector **f** was 77. Let the vector ${\bar{f}}_{l}={\left({\bar{a}}_{2l},\ldots ,{\bar{a}}_{Nl},{\bar{b}}_{2l},\ldots ,{\bar{b}}_{Nl},{\bar{c}}_{2l},\ldots ,{\bar{c}}_{Nl},{\bar{d}}_{1l},\ldots ,{\bar{d}}_{Nl}\right)}^{\text{T}}$ denote the average values of EFDs of six (dataset A) or four (dataset B) grains for the *l-*th accession of a rice germplasm collection. Based on the average values of EFDs, the average contour coordinates for the *l-*th accession can be calculated as

$${\bar{x}}_{l}(t)=\sum_{n=1}^{N}\left({\bar{a}}_{nl}\text{cos}\frac{2n\pi t}{T}+{\bar{b}}_{nl}\text{sin}\frac{2n\pi t}{T}\right)$$

and

$${\bar{y}}_{l}(t)=\sum_{n=1}^{N}\left({\bar{c}}_{nl}\text{cos}\frac{2n\pi t}{T}+{\bar{d}}_{nl}\text{sin}\frac{2n\pi t}{T}\right).$$

To visualize the grain shape variation observed in datasets A and B, we overlaid the average contour coordinates of all accessions variation in each dataset. The significance of among-accession variation against within-accession variation (i.e., variation among grains in each accession) was tested with the multivariate analysis of variance (MANOVA) using the ‘manova’ function in R [39].

### Genomic prediction of rice grain shape

We applied multiple methods to build a model that predicted EFDs based on genome-wide SNP marker genotypes. The first and second are methods that predict each coefficient of EFDs separately: we built a prediction model

$$f_{kl}=g(x),$$

where *f*_*kl*_ is the *k-*th entry of the vector **f**_*l*_ (i.e., the vector of EFDs of the *l-*th accession). **x** is a vector representing a genome-wide SNP marker genotype of the *l-*th accession. Each element of **x** had a value of 1 or -1 depending on the genotype of each marker. To build the prediction model *g*(**x**), we used ridge regression (RR) and non-linear kernel ridge regression (KRR). In the model building process, we used the function ‘kin.blup’ in the ‘rrBLUP’ package [40] in R. We calculated the realized additive relation matrix using the function ‘A.mat’ in the same package and used it as a kinship matrix used in the RR. In the KRR, we defined the kernel as

$$\kappa (x,\;x_{n})=\text{exp}\left(-h{\left(x-x_{n}\right)}^{2}\right),$$

where *h* is the bandwidth parameter. For this study, we chose $h=2/d_{m}^{2}$, where *d*_*m*_ is the median of the Euclidean distances of **x** among all pairs of accessions, as chosen by Crossa et al. [41].

The third and fourth are methods that predict all the coefficients of EFDs simultaneously: we built a prediction model,

$$f_{l}=g(x),$$

where **f**_*l*_ is the vector of EFDs of *l*th accession. For building the prediction model *g*(**x**), we used ordinary (i.e., linear) PLS regression (PLS) and nonlinear kernel PLS regression (KPLS). For the regression analysis, we used R scripts written by the first author. In the R scripts, we used the algorithm described by Rosipal and Trejo [42]. To determine the number of PLS components, we performed nested ten-fold cross-validation: we performed ten-fold cross-validation for determining the number of PLS components within each fold of the ten-fold cross-validation for evaluating the prediction accuracy. We set the maximum number of PLS components to 30. The Gaussian kernel used in KPLS was defined in the same way as KRR.

### Reconstruction of grain contours based on predicted EFDs

We drew the predicted grain shape based on the EFDs predicted from genome-wide SNP marker genotypes. Using Eqs. 1 and 2 and ignoring offsets of the centroid from the origin, i.e., *a*_0_ and *c*_0_, the prediction of *x* and *y* coordinates of the point at the length of the contour *t* from the starting point can be conducted based on the predicted EFDs as

$${\hat{x}}_{l}(t)=\sum_{n=1}^{N}\left({\hat{a}}_{ln}\text{cos}\frac{2n\pi t}{T}+{\hat{b}}_{ln}\text{sin}\frac{2n\pi t}{T}\right)$$

and

$${\hat{y}}_{l}(t)=\sum_{n=1}^{N}\left({\hat{c}}_{ln}\text{cos}\frac{2n\pi t}{T}+{\hat{d}}_{ln}\text{sin}\frac{2n\pi t}{T}\right)$$

where ${\hat{a}}_{ln}$, ${\hat{b}}_{ln}$, ${\hat{c}}_{ln}$ and ${\hat{d}}_{ln}$ are the predicted EFDs of the *l-*th accession. Setting *T* as 1.0 and *t* as 0, 0.01, …, 0.99, we calculated the coordinates of 100 points on a contour and drew the shape of the contour based on the calculated coordinate values.

### Evaluation of prediction accuracy

To evaluate the accuracy of the genomic prediction of grain shape, we performed cross-validation for which we calculated the squared prediction error of grain shape of each accession as follows. Integrating the displacement of *x* coordinates on a predicted contour from the corresponding *x* coordinates on an average contour in the *l-*th accession of a rice germplasm collection, we obtain the equations shown below.

$$\begin{array}{l} \int_{0}^{T}{\left[{\bar{x}}_{l}(t)-{\hat{x}}_{l}(t)\right]}^{2}dt\\ =\int_{0}^{T}{\left\{\sum_{n=1}^{N}\left[({\bar{a}}_{ln}-{\hat{a}}_{ln})\text{cos}\frac{2n\pi t}{T}+({\bar{b}}_{ln}-{\hat{b}}_{ln})\text{sin}\frac{2n\pi t}{T}\right]\right\}}^{2}dt\\ =\frac{T}{2}\sum_{n=1}^{N}\left[{\left({\bar{a}}_{ln}-{\hat{a}}_{ln}\right)}^{2}+{\left({\bar{b}}_{ln}-{\hat{b}}_{ln}\right)}^{2}\right] \end{array}$$

The integration of displacement in *y* coordinates of predicted and average contours in the *l-*th accession is calculable in the same way. Consequently, we can calculate the squared prediction error of grain shape of the *l-*th accession as the integration of displacement in both coordinates, as

$$\begin{array}{l} \int_{0}^{T}\left\{{\left[{\bar{x}}_{l}(t)-{\hat{x}}_{l}(t)\right]}^{2}+{\left[{\bar{y}}_{l}(t)-{\hat{y}}_{l}(t)\right]}^{2}\right\}dt\\ =\frac{T}{2}\sum_{n=1}^{N}\left[{\left({\bar{a}}_{ln}-{\hat{a}}_{ln}\right)}^{2}+{\left({\bar{b}}_{ln}-{\hat{b}}_{ln}\right)}^{2}+{\left({\bar{c}}_{ln}-{\hat{c}}_{ln}\right)}^{2}+{\left({\bar{d}}_{ln}-{\hat{d}}_{ln}\right)}^{2}\right]. \end{array}$$

The predicted residual sums of squares (PRESS) of grain shapes of all accessions was calculated as

$$\text{PRESS}=\frac{T}{2}\sum_{l=1}^{L}\sum_{n=1}^{N}\left[{\left({\bar{a}}_{ln}-{\hat{a}}_{ln}\right)}^{2}+{\left({\bar{b}}_{ln}-{\hat{b}}_{ln}\right)}^{2}+{\left({\bar{c}}_{ln}-{\hat{c}}_{ln}\right)}^{2}+{\left({\bar{d}}_{ln}-{\hat{d}}_{ln}\right)}^{2}\right].$$

The accuracy of predicted grain shapes was then measured as

$$Q^{2}=1-\text{PRESS}/\frac{T}{2}\sum_{l=1}^{L}\sum_{n=1}^{N}\left[{\left({\bar{a}}_{ln}-{\bar{\bar{a}}}_{n}\right)}^{2}+{\left({\bar{b}}_{ln}-{\bar{\bar{b}}}_{n}\right)}^{2}+{\left({\bar{c}}_{ln}-{\bar{\bar{c}}}_{n}\right)}^{2}+{\left({\bar{d}}_{ln}-{\bar{\bar{d}}}_{n}\right)}^{2}\right],$$ (3)

where ${\bar{\bar{a}}}_{k}$, ${\bar{\bar{b}}}_{k}$, ${\bar{\bar{c}}}_{k}$, and ${\bar{\bar{d}}}_{k}$ respectively denote the means of ${\bar{a}}_{lk}$, ${\bar{b}}_{lk}$, ${\bar{c}}_{lk}$, and ${\bar{d}}_{lk}$ over all accessions. The *Q* ^2^ represents the proportion of the variations explained by the prediction to the total variations in contour coordinates (i.e., shape variations). As described in the previous subsection, we set the value of *T* as 1.0 for calculating the squared prediction error of grain shape of each accession, PRESS and *Q* ^2^.

To evaluate the prediction accuracy based on the squared prediction error of grain shape of each accession and the *Q* ^2^ statistic, we conducted cross-validation of two types: leave-one-out cross-validation and ten-fold cross-validation. To evaluate the variation attributable to random splits of samples in the ten-fold cross-validation, we repeated ten-fold cross-validation 10 times on different splits of samples. In each replication, we used an identical random split of samples for all methods to enable the paired comparison of the prediction accuracy between methods. In ten-fold cross-validation, we can test the significance of difference between methods based on the prediction accuracy estimated in 10 replications. The significance of difference between methods was tested between RR and the other methods (i.e., KRR, PLS, and KPLS) with the Wilcoxon signed rank test that compared the matched pairs of a replication in the ten-fold cross-validation using the function ‘wilcox.exact’ in the R package ‘exactRankTests’. In the test, we regarded RR as a reference because it is the most common method used for genomic prediction and genomic selection.

## Results

### Rice grain shape variation delineated by EFDs

**Fig 1:**
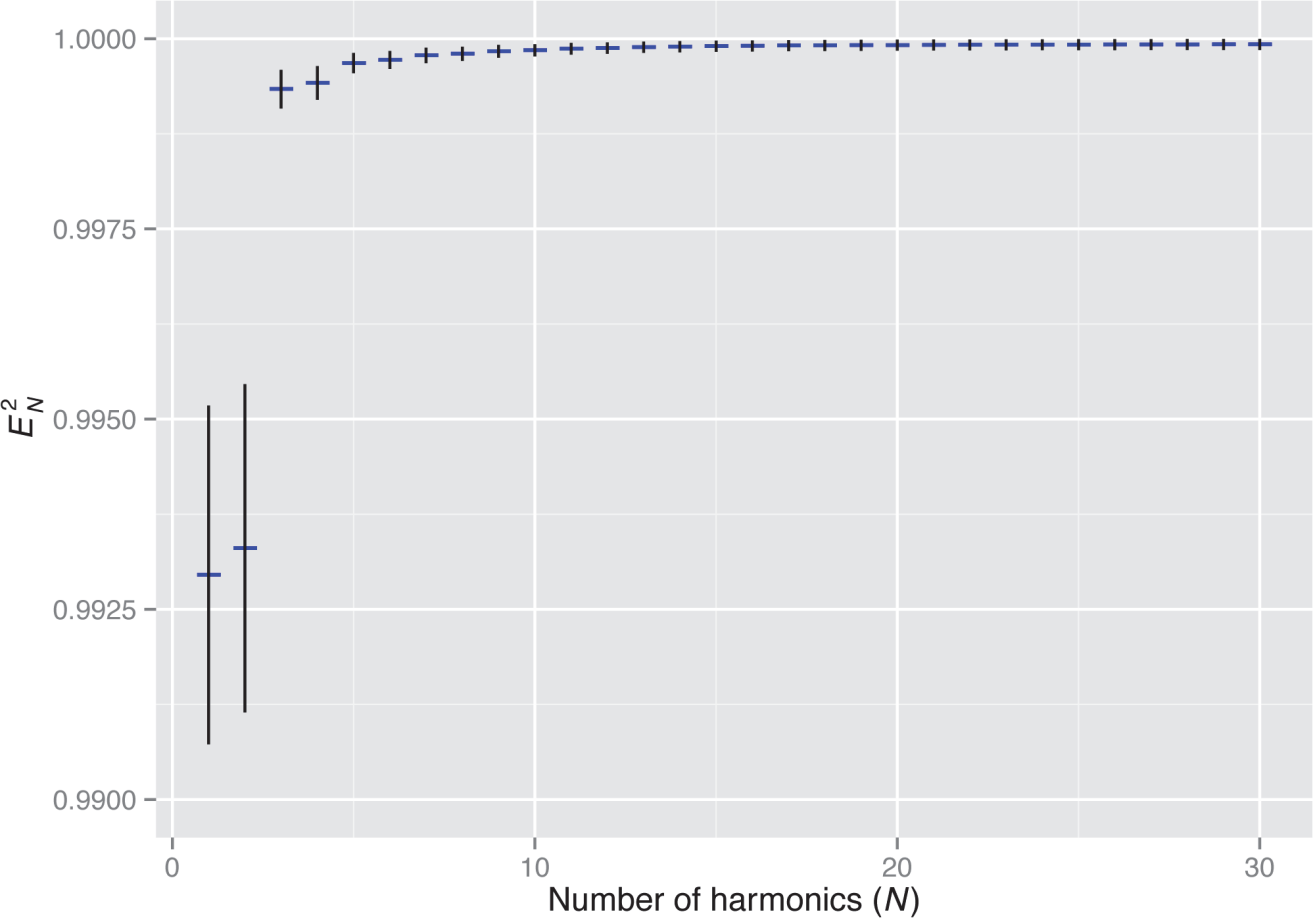
Accuracy of grain shape approximation using EFDs. The *x* and *y* axes show the number of harmonics of EFDs and the proportion of approximation error to the total grain shape variation, *E* ^*2*^_*N*_ (see text for details). Blue horizontal bars and black vertical bars show the mean and standard deviation of the *E* ^*2*^_*N*_ observed in 2,605 rice grains.

In this study, the contour coordinates of rice grains were extracted from digital images and then approximated with EFDs. We measured the contour shape of 2,605 grains. When the number of harmonics of EFDs was greater than 15, the error in the approximation with EFDs, $E_{N}^{2}$, was on average less than 0.01% of the total shape variation (Fig 1). For the following analyses, we used the first 20 harmonics of EFDs. Therefore, the variations in contour coordinates (i.e., shape variations) were almost exhaustively captured as the variation of EFDs.

**Fig 2:**
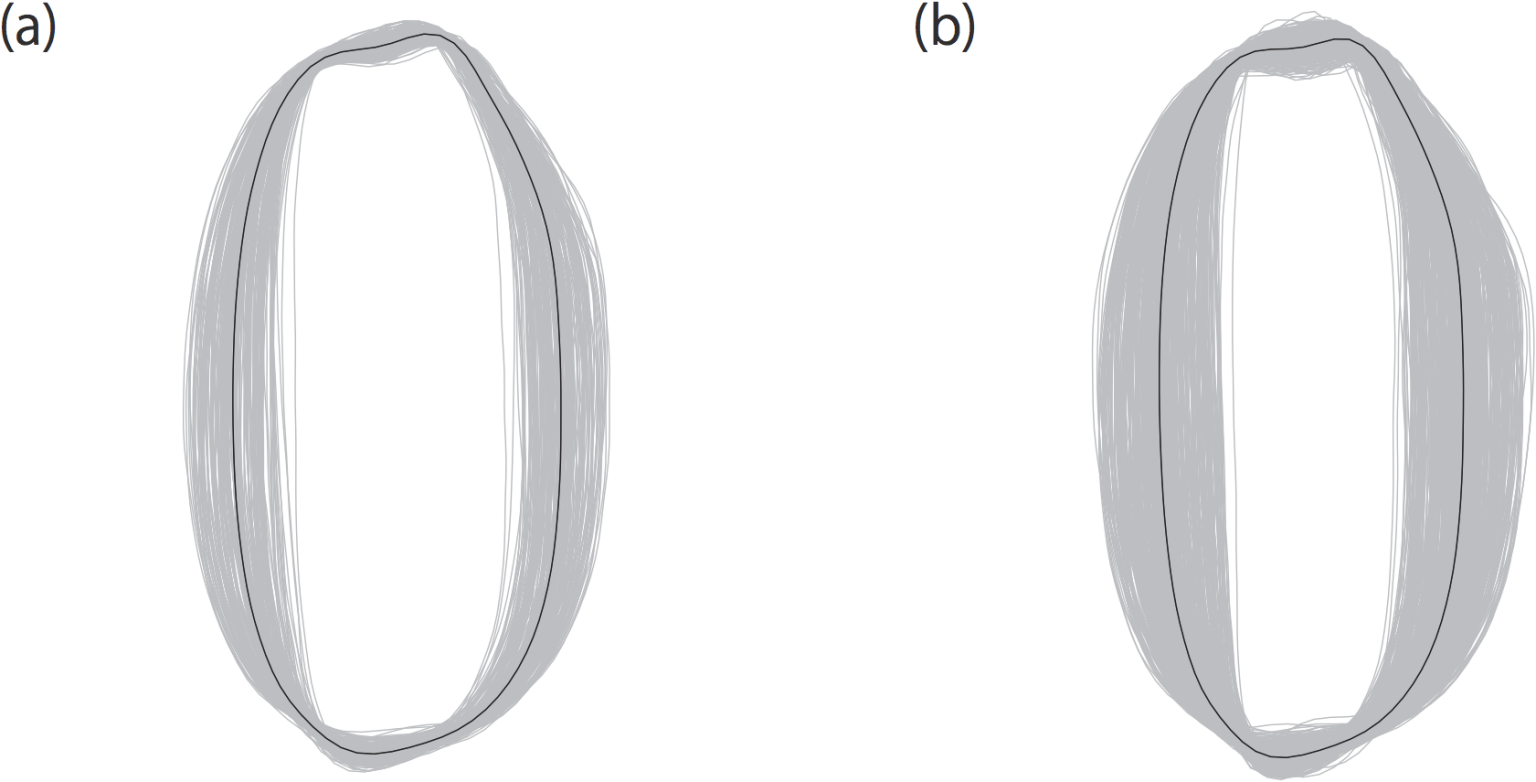
Grain shape variation observed in datasets A (a) and B (b). Average grain shapes of all accessions were overlaid. Thick contour lines represent the grain shape averaged over all accessions.

In Fig 2, the average grain shape of each accession was overlaid to visualize a grain shape variation among accessions. The among-accession variation was large in both datasets, and the length-to-width ratio of the grain was the major variation. A wider range of the variation was observed in dataset B than in dataset A (Fig 2). Especially, accessions having slender grain shape were included more frequently in dataset B than in dataset A. The MANOVA of EFDs revealed that the among-cultivar variation was significantly larger than the within-cultivar variation in both datasets (*F* = 1.52, *p* < 2.2 × 10^−16^ for dataset A and *F* = 2.05, *p* < 2.2 × 10^−16^ for dataset B), suggesting that variations in the average values of EFDs mostly reflect varietal differences of grain shapes.

### Prediction accuracy of rice grain shape

**Fig 3:**
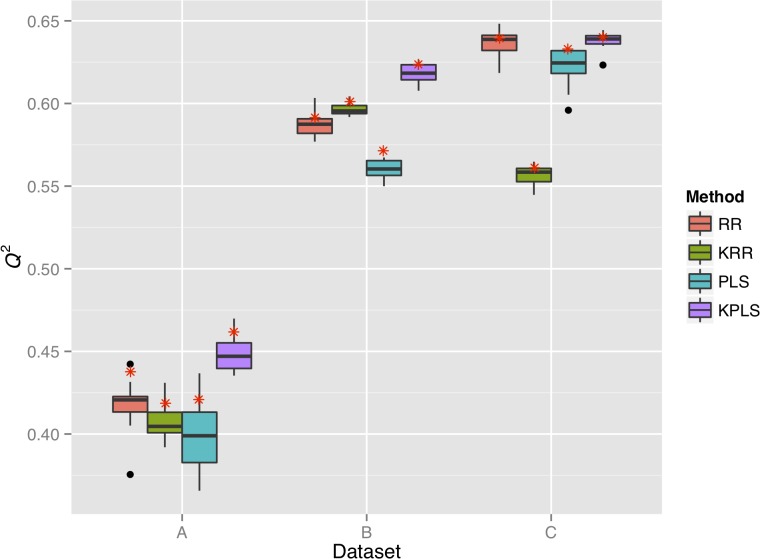
Prediction accuracy, *Q* ^2^, of rice grain shape in datasets A (a), B (b), and C (c). Each boxplot corresponds to a single method applied to a single dataset, and represents the range of *Q* ^2^ values obtained in the 10 replications of the ten-fold cross-validation. Red asterisks denote the *Q* ^2^ values obtained in the leave-one-out cross-validation.

For each of datasets A, B and C, we built a prediction model for rice grain shape using one of four different methods (i.e., RR, KRR, PLS, and KPLS), and evaluated the prediction accuracy of the models based on the *Q* ^2^ statistic (Fig 3). Based on the cross-validation of both types (i.e., ten-fold and leave-one-out cross-validation), the *Q* ^2^ showed the lowest values in dataset A among the datasets in all four methods. In datasets B and C, *Q* ^2^ showed similar values. In method-wise comparison, *Q* ^2^ was larger in dataset C than in dataset B, except for KRR. The *Q* ^2^ calculated via leave-one-out cross-validation was larger than the median of *Q* ^2^ in the 10 replications of ten-fold cross-validation, but it fell within a range of a variation observed in the 10 replications. The underestimation of *Q* ^2^ in the ten-fold cross-validation (or the overestimation of *Q* ^2^ in the leave-one-out cross-validation) was more pronounced in dataset A than in dataset B or C.

In dataset A, the *Q* ^2^ statistic was significantly different between RR on one hand and PLS (*p* < 0.05) and KPLS (*p* < 0.01) on the other in the ten-fold cross-validation. In fact, PLS was less accurate than RR, although KPLS was more accurate than RR (Fig 3). In leave-one-out cross-validation, KPLS showed the largest *Q* ^2^ among the four methods. In dataset B, the *Q* ^2^ statistics of RR and the other methods were significantly different (KRR, *p* < 0.01; PLS, *p* < 0.01; KPLS, *p* < 0.01). Results show that PLS was less accurate than RR, but KRR and KPLS were more accurate than RR. In leave-one-out cross-validation, KPLS showed the largest *Q* ^2^ among the four methods. In dataset C, the *Q* ^2^ statistic was significantly different between RR on one hand and KRR (*p* < 0.01) and PLS (*p* < 0.01) on the other. KRR and PLS were less accurate than RR. In the leave-one-out cross-validation, KPLS showed the largest *Q* ^2^ among the four methods. In the ten-fold cross-validation, however, the difference between RR and KPLS was not significant as described above. Next, we specifically assessed the prediction accuracy of KPLS evaluated via leave-one-out cross-validation.

**Fig 4:**
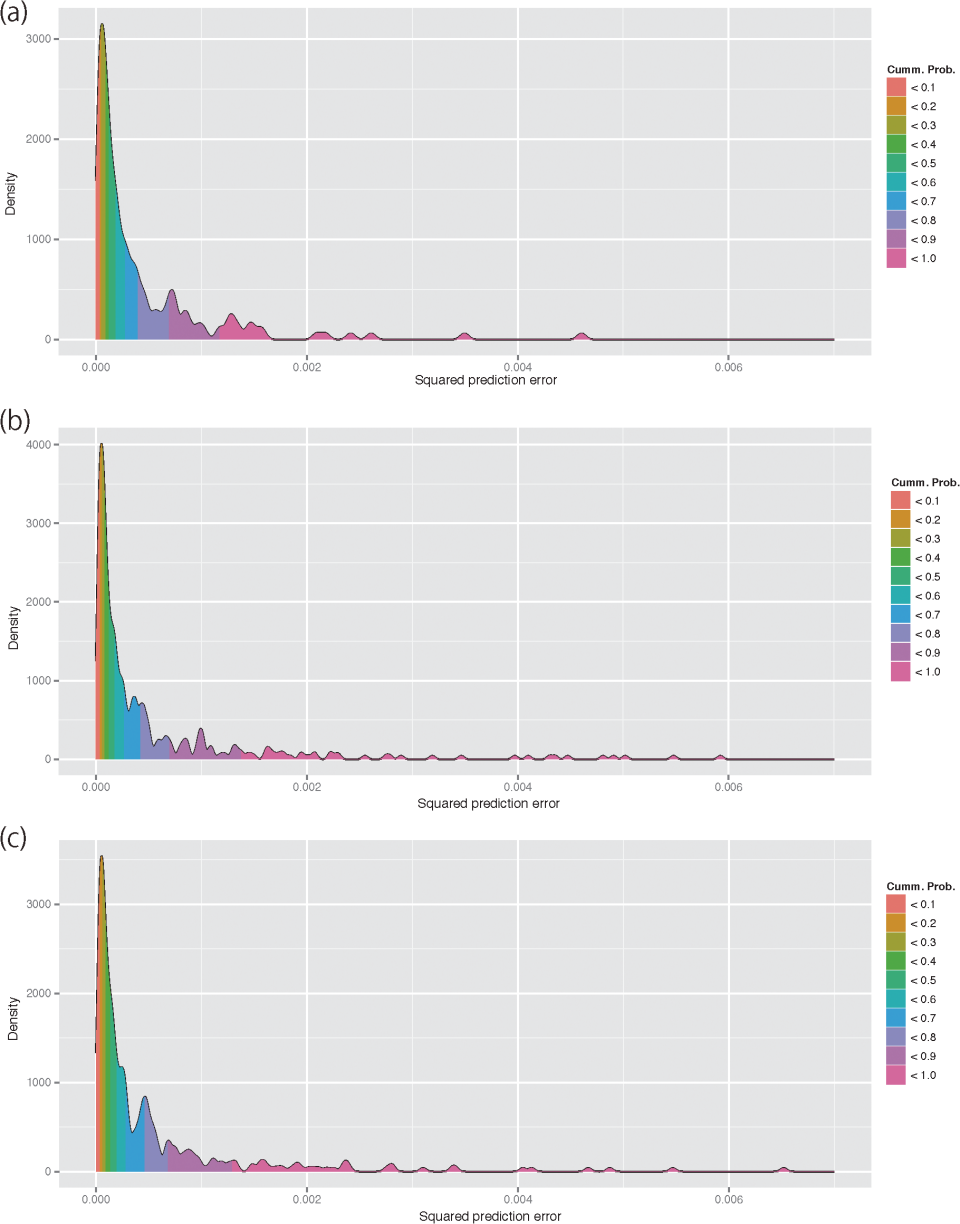
Empirical frequency distribution of squared prediction errors of all accessions in datasets A (a), B (b), and C (c). The empirical distribution was obtained using the algorithm of kernel density estimation. The distribution was filled with colors corresponding to the empirical cumulative probability of the squared prediction errors.

**Fig 5:**
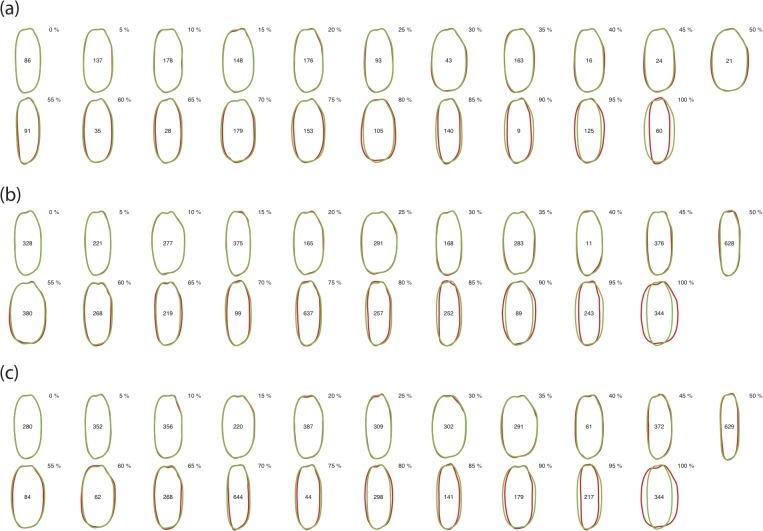
Rice grain shape predicted based on genome-wide SNP marker genotypes. A green contour line represents the predicted grain shape of each accession. An orange contour line represents the average grain shape of the accession. The grain shape prediction accuracy was evaluated via leave-one-out cross-validation.

Fig 4 depicts the frequency distribution in the squared prediction error of each accession. In all datasets, the distribution has a long right tail, but was strongly biased toward zero. In all datasets, the 70th percentile was in the head of the distribution, indicating that the prediction error was small in most (70%) accessions. Fig 5 depicts the predicted grain shape overlaid on the average grain shape of accessions that correspond to the 0th, 5th, 10th, …, 100th percentiles of the prediction errors. Accessions with prediction errors smaller than the median (i.e., 50th percentile) showed only slight difference between the predicted and average grain shapes. As portrayed in Fig 4, the difference between predicted and average grain shapes became readily apparent in the accessions with prediction errors larger than the 70th percentile. This trend was also observed in the grain shape prediction of other accessions (S1–S3 Figs.): in most (> 50%) accessions, the rice grain shape prediction based on genome-wide SNP marker genotypes was accurate at a practical level, whereas the prediction is difficult in some accessions. In accessions with larger prediction errors, thin and round types of rice grains were predicted as intermediate grains between thin and round types.

## Discussion

In this study, we assessed a method for the genomic prediction of the biological contour shape delineated by EFDs. Using this method, we can predict EFDs based on genome-wide marker genotype data. Predicted EFDs of each accession can be visualized as a predicted contour shape via inverse Fourier transformation. The visualization enables us to grasp shape variation intuitively, and makes the genomic prediction useful for select favorable shape based on genome-wide marker polymorphisms.

We delineated the rice grain shape with EFDs. Then we averaged EFDs over all grains measured for each accession. Based on the averaged EFDs, we built a model for predicting the grain shape of each accession. Then we evaluated the accuracy of the prediction model comparing the predicted EFDs with the averaged EFDs. Most (> 99.9%) of the variation in the rice grain shape was delineated by EFDs. Therefore, EFDs are good mediators for associating contour coordinate data to genome-wide marker data. In fact, EFDs can be standardized to size, rotation and the position of a starting point of the contour trace. Therefore, they are unaffected by these factors. Moreover, EFDs are independent of the number of sampling points (i.e., pixels) on the traced contour and represent the shape variation in uniform dimensions (77 dimensions in this study). These features make EFDs manageable mediators between contour coordinate data and genome-wide marker data. In this study, we performed MANOVA of EFDs. Results show that the among-accession variation of grain shape was highly significant. This result justifies that averaged EFDs are used in the construction of a prediction model and in the evaluation of prediction accuracy of the model.

The accuracy was lower in dataset A than in dataset B or C. The lower accuracy in dataset A has several possible causes. First, dataset A had smaller sample size (i.e., fewer accessions) than dataset B or dataset C. Small sample sizes for model buildings are well known to reduce the accuracy of the prediction model. The small sample size of dataset A might also cause the difference in the prediction accuracy estimated via the ten-fold cross-validation and leave-one-out cross-validation because the former cross-validation wastes 10% of the available data, whereas the latter wastes less than 1% (1 / 179) of the data. Second, the range of grain shape variation in dataset A was smaller than that in dataset B or dataset C. The *Q* ^2^ statistic is calculated as shown in Eq. 3. Therefore, it becomes smaller when the grain shape variation (the among-accession sum of squares of EFDs, i.e., the denominator of the second term) is smaller. In fact, the distribution of the squared prediction error of grain shape for each accession did not differ greatly between dataset A and dataset B or C (Fig 4). Third, the proportion of imputed genotypes was larger in dataset A (47.3%) than in dataset B or dataset C (3.1% and 4.3%). Wrongly imputed genotypes might cause inaccuracy in the prediction. A comparison between dataset B and dataset C showed that dataset C exhibited higher accuracy than dataset B did. The major difference between datasets B and C was the number of markers used for prediction. Linkage disequilibrium in cultivated rice was high especially in *japonica* rice [43]. The number of markers of dataset B, however, is regarded as insufficient for the accurate prediction of grain shape variation.

Among the four methods used for model building, KPLS showed the highest accuracy in all datasets. With KPLS, all EFDs were predicted simultaneously. EFDs have strong correlations among them. Therefore, the correlation structure in EFDs favors KPLS that models multiple independent variables (i.e., all EFDs) simultaneously. Ordinary PLS also models multiple dependent variables, but showed lower accuracy than RR which models a single dependent variable individually. The result suggests that nonlinear relations exist between multiple independent variables (i.e., EFDs) and dependent variables (i.e., marker genotypes), and that a nonlinear kernel approach is necessary for modeling the nonlinear relations. However, the relation between RR and KRR is opposite to the relation between KPLS and PLS: non-linear KRR showed lower accuracy than linear RR except in dataset B. The result suggests that the nonlinear relations between EFDs and marker genotypes become readily apparent when all EFDs are modeled simultaneously. The advantage of KPLS over RR was not significant in dataset C, which suggests that nonlinear relations between EFDs and marker genotypes are attributable to the insufficient sample size and marker density. Relations between prediction methods and prediction accuracy are so complex that the prediction accuracy of methods depends largely on the mode of inheritance of a target trait (i.e., degree of heritability, number of QTL, proportion of non-additive genetic variation), the size of a training population, and the linkage disequilibrium structure [44]. Additional studies must be conducted to investigate factors causing the nonlinear relations, including nonlinear genetic effect such as epistasis.

The genomic prediction of plant organ shape enables us to select plants with favorable shape based on genome-wide marker polymorphisms (i.e., genomic selection) without observing their actual shape via field experiments. Many benefits of the genomic selection relative to conventional phenotypic selection have been suggested [18, 19]. As viewed from the perspective of genetic improvement of plant organ shape, some of the great benefits of genomic selection are the following. (1) The shape of agriculturally important organs such as fruits and seeds is expressed after the reproductive phase. Using genomic prediction, plants with favorable shapes can be selected in the early vegetative phase (i.e., seedling development). This benefit is of great importance particularly for species that have long vegetative phase, such as fruit trees [24]. (2) In the genetic improvement of crop organ shape, a required selection scheme is usually not simple directional selection because the optimum shape is based on well balanced multiple factors in general. In such a trait, ordinary MAS might not be an appropriate selection method because of its simplicity. Using selection based on the genomic prediction of crop organ shape, it is possible to confirm the predicted shapes of selection candidates visually and to select genotypes having favorable shapes. Consequently, the method proposed in this study presents great advantages over ordinary MAS. Using the method proposed by Iwata et al. [45], it is possible to predict the segregation pattern of a target trait in a progeny population based on a genomic selection prediction model. The method will facilitate the selection of good parental combinations that have high potential to produce progenies with optimum shape.

Quantitative shape measurement based on EFDs has been applied to the quantitative genetic analyses of biological shape. Based on EFDs of biological contours, QTLs controlling biological shape variation have been detected via bi-parental QTL mapping [35–38, 46, 47] and genome-wide association studies [13, 48]. Successful application of EFDs to the quantitative genetic analyses of biological shape demonstrates that EFDs can delineate the continuous shape variation accurately and that they can capture genetic variation underlying the phenotypic variation of biological shape. The achievements also suggest that the genomic prediction of biological shape proposed in this paper is applicable to other species as well. Currently, high-throughput genotyping technologies are available for a range of organisms [49]. They might facilitate the genomic prediction of biological shapes in various species. Further studies are warranted to evaluate the efficiency of the proposed method in the genomic prediction of more complex biological shape variation observed in other species.

Biological shape is influenced by environmental and genetic factors. In this study, we evaluated the prediction accuracy of the proposed method with two independent datasets. In neither dataset, however, was there environmental replication. When the influence of environment factors is strong, the prediction accuracy of a model built in one environment (e.g., location, year) might become worse in another environment [50]. This is an issue in genomic selection, but it has not been addressed well in the literature [51]. Few studies have examined multi-environmental evaluation of plant shape variation [52–55]. Therefore, more studies must be done to investigate the influence of environmental factors on the accuracy of genomic prediction of plant shape.

This study revealed kernel PLS regression as the most efficient method to build a model for predicting genetic values in a high-dimensional trait, rice grain shape, based on genome-wide SNP marker genotypes. The result suggests the potential of kernel PLS regression for the genomic prediction of other high-dimensional traits. Recently, high-throughput phenotyping systems have been developed to facilitate the measurement of phenotypic variation and to relieve a severe obstacle to ‘omics’ studies, i.e., so-called ‘phenotyping bottleneck’ [56]. In the high-throughput phenotyping systems, various novel technologies, including image analysis, are used. They generate large amounts of high-dimensional phenotypic data. As suggested in this study, the kernel PLS regression seems useful for modeling the relation between a high-dimensional quantitative trait and genome-wide marker polymorphisms. No report in the literature has described a study in which kernel PLS regression was applied to model building in genomic selection. Additional studies must be conducted to evaluate the potential of the kernel PLS regression for the genomic selection of high-dimensional traits.

## Conclusions

As described herein, we proposed a method for predicting the biological contour shape based on genome-wide SNP markers. Results obtained from an empirical study using rice germplasm accessions suggested the potential of the proposed method in rice grain shape prediction. The method enables application of genomic selection to rice grain shape improvement. EFDs have been applied to genetic analyses of biological shape. This method is expected to be useful for other species as well. As described herein, we proposed the use of kernel PLS regression to predict high-dimensional EFDs, with an approach that might be useful also for the genomic prediction of a high-dimensional trait obtained using high-throughput phenotyping technology.

## Supporting Information

S1 Dataset Genome-wide SNP marker data and elliptic Fourier descriptor data in datasets A, B, and C are provided in the zip file. The zip file includes two directories: Directory “GenotypeScore” contains SNP genotype scores after imputation of missing genotypes, and directory “FourierDescriptor” contains varietal averages of elliptic Fourier descriptors.
(ZIP)

S1 Fig Predicted and average grain shapes of rice germplasm accessions in dataset A. A green contour line represents the predicted grain shape of each accession. An orange contour line represents the average grain shape of the accession. The grain shape prediction accuracy was evaluated via leave-one-out cross-validation.
(TIF)

S2 Fig Predicted and average grain shapes of rice germplasm accessions in dataset B. A green contour line represents the predicted grain shape of each accession. An orange contour line represents the average grain shape of the accession. The grain shape prediction accuracy was evaluated via leave-one-out cross-validation.
(TIF)

S3 Fig Predicted and average grain shapes of rice germplasm accessions in dataset C. A green contour line represents the predicted grain shape of each accession. An orange contour line represents the average grain shape of the accession. The grain shape prediction accuracy was evaluated via leave-one-out cross-validation.
(TIF)

S1 Table Accessions in rice germplasm at the National Institute of Agrobiological Sciences (NIAS) Genebank, which were involved in dataset A. (PDF)

S2 Table Accessions in Genetic Stocks—Oryza (GSOR) involved in datasets B and C. (PDF)
