# Supplementary figures and images for: Genomic Prediction of Biological Shape: Elliptic Fourier Analysis and Kernel Partial Least Squares (PLS) Regression Applied to Grain Shape Prediction in Rice (Oryza sativa L.)
Source: PLoS One. 2015 Mar 31;10(3):e0120610. doi: 10.1371/journal.pone.0120610 (PMC4380318; doi:10.1371/journal.pone.0120610)

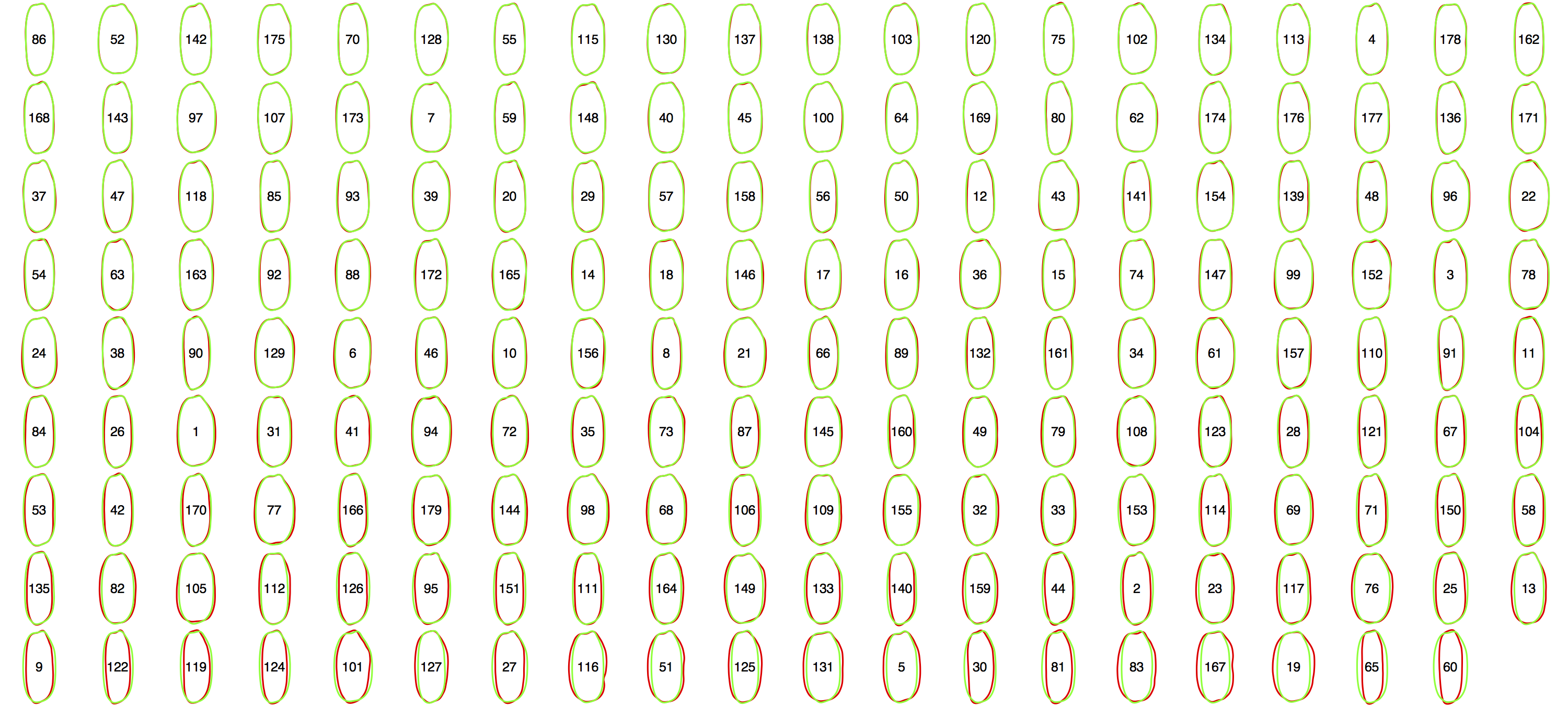

Supplement: S1 Fig — A green contour line represents the predicted grain shape of each accession. An orange contour line represents the average grain shape of the accession. The grain shape prediction accuracy was evaluated via leave-one-out cross-validation. (TIF) [file pone.0120610.s002.tif]

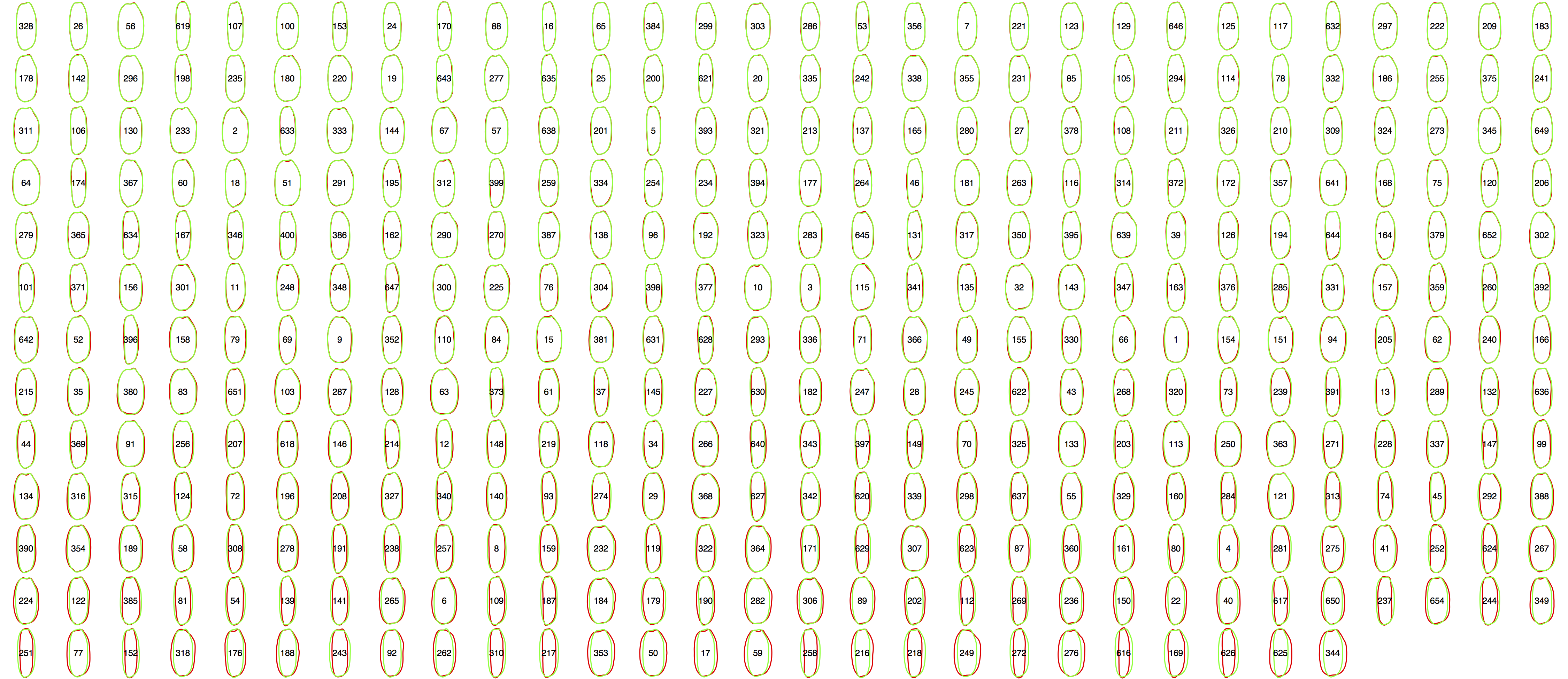

Supplement: S2 Fig — A green contour line represents the predicted grain shape of each accession. An orange contour line represents the average grain shape of the accession. The grain shape prediction accuracy was evaluated via leave-one-out cross-validation. (TIF) [file pone.0120610.s003.tif]

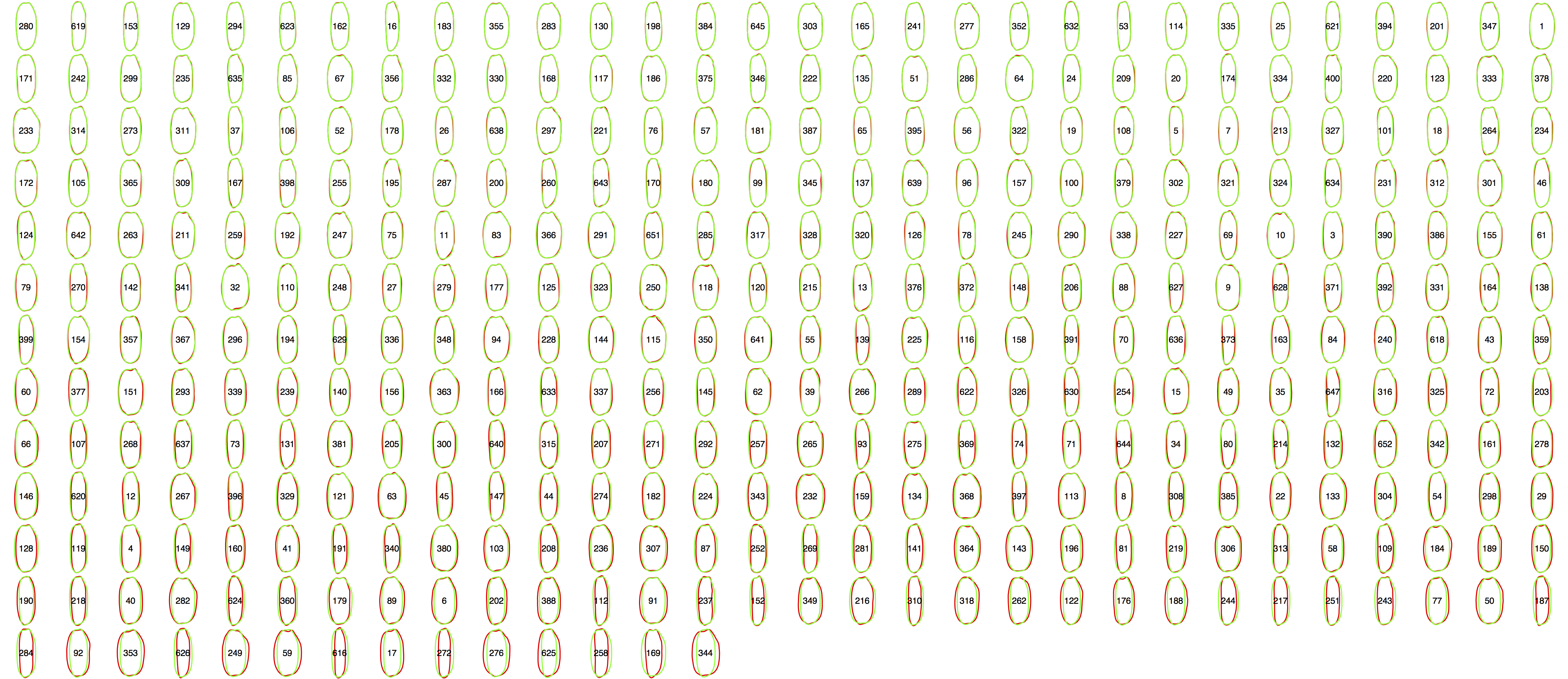

Supplement: S3 Fig — A green contour line represents the predicted grain shape of each accession. An orange contour line represents the average grain shape of the accession. The grain shape prediction accuracy was evaluated via leave-one-out cross-validation. (TIF) [file pone.0120610.s004.tif]
