# Supplementary material for: Genomic Prediction of Biological Shape: Elliptic Fourier Analysis and Kernel Partial Least Squares (PLS) Regression Applied to Grain Shape Prediction in Rice (Oryza sativa L.)
Source: PLoS One. 2015 Mar 31;10(3):e0120610. doi: 10.1371/journal.pone.0120610 (PMC4380318; doi:10.1371/journal.pone.0120610)
Supplement: S1 Table — (PDF) [file pone.0120610.s005.pdf]

Table S1. Accessions in rice germplasm at the National Institute of Agrobiological Sciences (NIAS) Genebank, which were involved in dataset A

JP.ID*	Name	JP.ID	Name
71249	91-382	84983	CS-S4
11930	Aijiaonante	83160	Dabaigu
43474	Aijiaonuo	79697	Dahonggu
NA**	Akage	11619	Daidantou
NA	Akihikari	83139	Dalibaigu
70701	Amane	84313	Dangrey
104588	Angpor Sang San	12426	Davao 1
54300	Anjana Dhan	147493	Deegeowoogen
54581	ARC 11094	NA	Deejiaohualuo
54610	ARC 5955	11828	Deng Pao Zhai (Toufutsusai)
54631	ARC 6565	54518	Dhalashaita
54573	ARC 7047	42944	Dianyu 1
54575	ARC 7260	37948	Dinalaga
54633	ARC 7286	81944	Dumja Kaap
54576	ARC 7291	NA	Fujisaka 5
54634	ARC 7323	54586	Gambir
54635	ARC 7336	NA	Ginbozu
NA	Asahi	14113	Godawee
71239	Asu	11987	Guangluai 4
54551	Aus 230	71244	Hasu
70622	Aus 32	54373	Heipigu
70620	Aus 47	14103	Hetadawee
70541	Badari Dhan	NA	Hong Cheuh Zai
12422	Basilanon	71375	Honggu
105329	Bayuenuo	37735	Hongxienuo
80448	Bei Khe	14109	Illan Kalagan
NA	Bie Blau	71274	Ippa
12425	Binatangan 2	12456	IR 29
12484	Bincol	180265	IR 58
12719	Bodat Mayang	70452	Jadu
82004	Bunt Kardu	14836	Jaguary
12486	Calotoc	71261	Jarjan
12416	Calutos	14030	Jena 035
86156	Cha Sen Lun	54272	Jhimma
12932	Chinsurah Boro 2	NA	Jhona 2
53955	Chinya	NA	Jikkoku
11611	Chuumoushi	79691	Jinguoin
12907	Co 13	11814	Junsouhaku

JP.ID	Name	JP.ID	Name
NA	Kahei	80450	Neang Chhouk
11872	Kakouhaku	NA	Nepal 1
54283	Kalo Dhan	NA	Nepal 18
54630	Kaloshahaita	NA	Nepal 555
14070	Kaluheenati	85148	Nepal 8
NA	Kameji	77338	Nepale Jaran
11866	Kamochi Kantonfukinsan	86151	Ngo Luu
44977	Kampar	NA	Nipponbare
205306	Kasalath	54372	Niuyanggu 1
11633	Keiboba	NA	Norin 1
76885	Ketan Gading	NA	Norin 29
104129	Khao Hang	NA	Norin 8
78799	Khao Kwe Lan	83126	Oazalahy
12568	Khao Nok	NA	Oba
83195	Khao Vay Deng	NA	Ochikara
86166	Khau Mac Kho	70700	Pachai Perumal
86141	Khau Tan Chiem	181273	Padi Bulu
86139	Khau Van Lanh	12721	Padi Kenikir Puti
100783	Khemjya	103839	Padi Kuning
NA	Kinandang Puti	74372	Padi Perak
NA	Kinuhikari	101011	Peam Chiker
100802	Kochum	12418	Pinulupot 1
NA	Koshihikari	74384	Puluik Arang
11597	Lalahpak	12912	Pusur
12571	Lep Xang	53938	Puteh/Rubon(H)
100350	Linjawan(H)	106328	Py Khao
NA	Liuzhoubaoyazao	12170	Qingyu (Seiyu)
13277	Local Basmati	13178	Rambhog
12853	Ma sho	13672	Ratul
77185	Manan Elatla	NA	Reiho
74610	Maninjau	NA	Reimei
11559	Manri 2	109700	Rexmont
NA	Masumikir	14044	Seenady
37823	Milyang 23	NA	Sekiyama
11777	Moch Ine Sukensan	11555	Shinha Sen
81699	Moe Ma Kha	NA	Shinriki
NA	Muha	54535	Shoni
13351	Naba	11605	Shoutan Zairai

JP.ID	Name	JP.ID	Name
11831	Shuusoushu		
80366	Shwe Nang Gyi		
12723	Siampang		
103853	Sikasunbo		
181302	Sirendah Putih		
12887	Surjamukhi		
12485	Tadukan		
12276	Taichung 65		
11647	Taiwan Sou		
71279	Thapa Chinni		
81972	Tima		
11222	Toboshi		
11821	Totou		
70609	Tupa 121-3		
70616	Tupa729		
71954	Turakin		
11595	Ubaisen		
4656	Urasan 1		
54647	Vandaran		
77231	Vary Futsi		
83162	Wahuigu		
4375	Wase Sekitori F		
14286	Wzbeuskij 2		
86100	Xiaobaigu		
NA	Xiligu		
NA	Xuanchangmi		
11731	Yousentou 29		

*Identification number of rice germplasm at the NIAS Genebank

**Non-available
