# Supplementary material for: Genomic Prediction of Biological Shape: Elliptic Fourier Analysis and Kernel Partial Least Squares (PLS) Regression Applied to Grain Shape Prediction in Rice (Oryza sativa L.)
Source: PLoS One. 2015 Mar 31;10(3):e0120610. doi: 10.1371/journal.pone.0120610 (PMC4380318; doi:10.1371/journal.pone.0120610)
Supplement: S2 Table — (PDF) [file pone.0120610.s006.pdf]

Table S2. Accessions in Genetic Stocks - Oryza (GSOR) involved in datasets B and C

GSOR*	Name	Datasets**	GSOR	Name	Datasets
301001	Agostano	B, C	301047	Early Wataribune	B, C
301002	Aichi Asahi	B	301048	Eh Ia Chiu	B, C
301003	Ai-Chiao-Hong	B, C	301049	Firooz	B, C
301004	NSF-TV 4	B, C	301050	Fortuna	B, C
301005	NSF-TV 5	B, C	301051	Gerdeh	B, C
301006	ARC 7229	B, C	301052	Geumobyeo	B, C
301007	Arias	B, C	301053	NSF-TV 57	B, C
301008	Asse Y Pung	B, C	301054	Ghati Kamma Nangarhar	B, C
301009	Baber	B, C	301055	Gogo Lempuk	B, C
301010	Baghlani Nangarhar	B, C	301056	Gotak Gatik	B, C
301382	Baguamon 15	B, C	301057	Guan-Yin-Tsan	B, C
301011	Basmati	B, C	301386	Gyehwa 3	B, C
301012	NSF-TV 13	B, C	301387	Haginomae Mochi	B, C
301383	Beonjo	B, C	301388	Heukgyeong	B, C
301014	Bico Branco	B, C	301058	Honduras	B, C
301015	Binulawan	B, C	301059	Hsia Chioh Keh Tu	B, C
301016	BJ 1	B, C	301060	Hu Lo Tao	B, C
301017	Black Gora	B, C	301062	IAC 25	B, C
301018	Blue Rose	B, C	301063	Iguape Cateto	B, C
301020	Caawa/Fortuna 6-103-15	B, C	301064	IR 36	B, C
301022	Carolina Gold	B, C	301065	IR 8	B, C
301023	Carolina Gold	B, C	301066	IRAT 177	B, C
301024	Carolina Gold Sel	B, C	301067	IRGA 409	B, C
301025	NSF-TV 27	B, C	301068	Jambu	B, C
301026	Champa Tong 54	B	301069	Jaya	B, C
301027	Chau	B, C	301070	JC149	B, C
301030	Chodongji	B, C	301071	Jhona 349	B, C
301032	NSF-TV 34	B, C	301072	Jouiku 393G	B, C
301033	CO18	B, C	301073	K 65	B, C
301035	Cuba 65	B, C	301074	Kalamkati	B, C
301037	NSF-TV 39	B, C	301075	Kamenoo	B, C
301038	Dam	B, C	301076	Kaniranga	B, C
301039	Darmali	B, C	301077	Kasalath	B, C
301040	Dee Geo Woo Gen	B, C	301079	Keriting Tingii	B, C
301041	Dhala Shaitta	B, C	301080	Khao Gaew	B, C
301042	Dom Sufid	B, C	301081	NSF-TV 89	B, C
301043	Dourado Agulha	B, C	301083	Kibi	B, C
301045	DV85	B, C	301084	Kinastano	B, C
301046	DZ78	B, C	301085	Kitrana 508	B, C

GSOR	Name	Datasets	GSOR	Name	Datasets
301086	Koshihikari	B, C	301132	Seratoes Hari	B, C
301088	KU115	B, C	301133	Shai-Kuh	B, C
301091	LAC 23	B, C	301134	Shinriki	B, C
301092	Lacrosse	B, C	301135	Shoemed	B, C
301093	Lemont	B, C	301136	Short Grain	B, C
301095	Luk Takhar	B, C	301137	Shuang-Chiang	B, C
301097	Mehr	B, C	301138	Sinampaga Selection	B, C
301098	Ming Hui	B, C	301139	Sintane Diofor	B, C
301099	NSF-TV 107	B, C	301140	Sinaguing	B, C
301100	Moroberekan	B, C	301141	Sultani	B, C
301101	MTU9	B, C	301142	Suweon	B, C
301102	Mudgo	B, C	301143	T 1	B, C
301104	N12	B, C	301144	T26	B, C
301105	Norin 20	B, C	301145	Ta Hung Ku	B, C
301106	Nova	B, C	301146	Ta Mao Tsao	B, C
301107	NPE 835	B, C	301147	Taichung Native 1	B, C
301108	NSF-TV 116	B, C	301148	Tainan Iku 487	B, C
301109	O-Luen-Cheung	B, C	301149	Taipei 309	B, C
301110	Oro	B, C	301150	Tam Cau 9A	B, C
301111	Oryzica Llanos 5	B, C	301151	NSF-TV 160	B, C
301112	OS6	B, C	301152	TeQing	B, C
301113	Ostiglia	B, C	301153	TKM6	B, C
301114	Padi Kasalle	B, C	301154	Taducan	B, C
301115	Pagaiyahan	B, C	301155	Tondok	B, C
301390	Pankhari 203	B, C	301156	Trembese	B, C
301116	Pao-Tou-Hung	B, C	301157	Tsipala 421	B, C
301117	Pappaku	B, C	301158	B6616A4-22-Bk-5-4	B, C
301119	Pato De Gallinazo	B, C	301159	Vary Vato 462	B, C
301120	Peh-Kuh	B, C	301160	WC 6	B, C
301121	Peh-Kuh-Tsao-Tu	B, C	301161	Wells	B, C
301122	Phudugey	B, C	301162	ZHE 733	B, C
301123	Rathuwee	B, C	301163	Zhenshan 2	B, C
301124	Rikuto Kemochi	B, C	301165	Azucena	B, C
301125	Romeo	B, C	301167	583	B, C
301126	RT 1031-69	B, C	301168	Feb.68	B, C
301128	RTS14	B, C	301169	ARC 6578	B, C
301129	RTS4	B, C	301170	Bellardone	B, C
301130	S4542A3-49B-2B12	B, C	301171	Benllok	B, C
301131	Saturn	B, C	301172	Bergreis	B, C

GSOR	Name	Datasets	GSOR	Name	Datasets
301173	Blue Rose Supreme	B, C	301221	Hunan Early Dwarf No. 3	B, C
301174	Boa Vista	B, C	301222	Shangyu 394	B, C
301175	Bombon	B, C	301223	Sung Liao 2	B, C
301177	Bul Zo	B, C	301224	Aijiaonante	B, C
301178	C57-5043	B, C	301225	Sze Guen Zim	B, C
301179	Coppocina	B, C	301226	WC 521	B, C
301180	Criollo La Fria	B, C	301227	Estrela	B, C
301181	Delrex	B, C	301228	WAB 56-104	B
301182	Dom Zard	B, C	301229	WAB 502-13-4-1	B, C
301183	Erythroceros Hokkaido	B, C	301230	WAB 501-11-5-1	B, C
301185	HG 24	B, C	301231	ECIA76-S89-1	B, C
301186	IRAT 13	B, C	301232	27	B, C
301187	JM70	B, C	301233	Tropical Rice	B, C
301189	Leah	B, C	301234	Arabi	B, C
301191	P 737	B, C	301235	Sab Ini	B, C
301192	Pate Blanc Mn 1	B, C	301237	Desvauxii	B, C
301193	Pratao	B, C	301238	Caucasica	B, C
301194	Radin Ebos 33	B, C	301239	Pirinae 69	B, C
301196	Rinaldo Bersani	B, C	301240	Bulgare	B, C
301197	Rojofotsy 738	B, C	301241	H256-76-1-1-1	B, C
301198	Sigadis	B, C	301242	Djimoron	B, C
301199	SLO 17	B, C	301244	Hon Chim	B, C
301200	Tchibanga	B, C	301245	Pai Hok Glutinous	B, C
301201	Thavalu	B	301246	Romanica	B, C
301202	Tokyo Shino Mochi	B, C	301247	Agusita	B, C
301204	WC 3397	B, C	301248	Tia Bura	B, C
301205	WC 4419	B, C	301249	Sadri Tor Misri	B, C
301206	WC 4443	B, C	301250	NSF-TV 260	B, C
301207	Yabani Montakhab 7	B, C	301252	Halwa Gose Red	B, C
301208	YRL-1	B, C	301253	Maratelli	B, C
301209	PI 298967-1	B, C	301254	Baldo	B, C
301210	Nucleoryza	B, C	301255	Vialone	B, C
301211	Azerbaidjanica	B, C	301256	Hiderisirazu	B, C
301212	Sadri Belyi	B, C	301257	Hatsunishiki	B, C
301213	Paraiba Chines Nova	B, C	301258	Vavilovi	B, C
301215	Karabaschak	B, C	301259	Sundensis	B, C
301216	Biser 1	B, C	301260	Osogovka	B, C
301218	Riz Local	B, C	301261	M. Blatec	B, C
301219	CA 902/B/2/1	B, C	301262	923	B, C

GSOR	Name	Datasets	GSOR	Name	Datasets
301263	Varyla	B, C	301305	Dawebyan	B, C
301264	Padi Pagalong	B, C	301306	DD 62	B, C
301265	Sri Malaysia Dua	B, C	301307	DJ 123	B, C
301266	Kaukau	B, C	301308	DJ 24	B, C
301267	Gambiaka Sebela	B, C	301310	DM 43	B, C
301268	C1-6-5-3	B, C	301311	DM 56	B, C
301269	Kon Suito	B, C	301312	DM 59	B, C
301270	Saku	B, C	301313	DNJ 140	B, C
301271	Patna	B, C	301314	DV 123	B, C
301272	Triomphe Du Maroc	B, C	301315	EMATA A 16-34	B, C
301273	Chibica	B, C	301316	Ghorbhai	B, C
301274	IR-44595	B, C	301317	Goria	B, C
301275	Tox 782-20-1	B, C	301318	Jamir	B, C
301276	IITA 135	B, C	301319	Kachilon	B, C
301277	Zerawchanica Karatalski	B, C	301320	Khao Pahk Maw	B, C
301279	Lusitano	B, C	301321	Khao Tot Long 227	B, C
301280	Amposta	B, C	301322	KPF-16	B, C
301281	Toploea 70/76	B, C	301323	Leuang Hawn	B, C
301282	Stegaru 65	B, C	301324	Lomello	B, C
301283	TOg 7178	B, C	301325	Okshitmayin	B, C
301284	SL 22-613	B, C	301326	Paung Malaung	B, C
301286	Dosel	B, C	301327	Sabharaj	B, C
301287	Bahia	B, C	301328	Sitpwa	B, C
301288	LD 24	B, C	301329	Yodanya	B, C
301289	SML 242	B, C	301330	Berenj	B, C
301290	Sml Kapuri	B, C	301331	Shirkati	B, C
301291	Melanotrix	B, C	301332	Cenit	B, C
301292	WIR 3039	B, C	301333	Victoria F.A.	B, C
301293	Kihogo	B, C	301334	Habiganj Boro 6	B, C
301294	519	B, C	301335	DZ 193	B, C
301296	WIR 3764	B, C	301336	Karkati 87	B, C
301297	Uzbekskij 2	B, C	301337	Creole	B, C
301298	Llanero 501	B, C	301338	China 1039	B, C
301299	Manzano	B, C	301339	Chang Ch'Sang Hsu Tao	B, C
301300	R 101	B, C	301340	Ligerito	B, C
301301	56-122-23	B, C	301393	Guatemala 1021	B, C
301302	Aswina 330	B, C	301341	ARC 10376	B, C
301303	BR24	B, C	301342	BALA	B
301304	CTG 1516	B, C	301343	ASD 1	B, C

GSOR	Name	Datasets	GSOR	Name	Datasets
301344	JC 117	B, C	301404	Pecos	B, C
301345	9524	B, C	301405	Rosemont	B, C
301347	Surjamkuhi	B, C	301406	Jasmine85	B, C
301348	PTB 30	B, C	301402	LaGrue	B, C
301350	Edomen Scented	B, C	301418	Bengal	B, C
301351	Rikuto Norin 21	B, C	301407	Shufeng 121-1655	B, C
301352	Shirogane	B, C	301408	Kaybonnet	B, C
301353	Kiuki No. 46	B, C	301419	Katy	B, C
301354	Sanbyang-Daeme	B, C	301420	C101A51	B, C
301355	Deokjeokjodo	B, C	301421	Early	B, C
301356	Sathi	B, C	301409	Jefferson	B, C
301358	Santhi Sufaid	B, C	301410	Panda	B, C
301359	Sufaid	B, C	301411	Saber	B, C
301360	Lambayeque 1	B, C	301412	Dragon Eyeball 100	B
301396	WC 2810	B, C	301413	Francis	B, C
301361	Breviaristata	B, C	301414	Jing 185-7	B, C
301362	PR 304	B, C	301415	Rondo (4484-1693)	B, C
301363	Kalubala Vee	B, C	312001	Azucena	B, C
301364	Wanica	B, C	312002	Sadu Cho	B, C
301365	Tainan-Iku No. 512	B, C	312003	N 22	B, C
301366	325	B, C	312004	Moroberekan	B, C
301367	318	B, C	312005	Nipponbare	B, C
301368	Nira	B, C	312006	Dom Sufid	B, C
301369	Palmyra	B, C	312007	Tainung 67	B, C
301370	M-202	B, C	312008	Zhenshan 97B	B, C
301371	Nortai	B, C	312009	Minghui 63	B, C
301373	CI 11026	B, C	312010	IR64	B, C
301374	Della	B, C	312011	M-202	B, C
301375	Edith	B, C	312012	Swarna	B
301376	La 110	B	312013	Cypress	B, C
301377	Lady Wright Seln	B, C	312015	FR13A	B
301378	OS 6 (WC 10296)	B, C	312016	Aswina	B
301379	Cocodrie	B, C	312017	Dular	B, C
301380	Cybonnet	B, C	312018	Li-Jiang-Xin-Tuan-Hei-Gu	B, C
301399	Nov.93	B, C	312020	Pokkali	B
301381	Spring	B, C			
301400	Yang Dao 6	B, C			
301416	RT0034	B, C			
301417	MCR010277	B			

*Identification number of the GSOR

**Datasets in which each accession was involved
